# Neuroendocrine Tumors Show Altered Expression of Chondroitin Sulfate, Glypican 1, Glypican 5, and Syndecan 2 Depending on Their Differentiation Grade

**DOI:** 10.3389/fonc.2014.00015

**Published:** 2014-02-07

**Authors:** Olivia García-Suárez, Beatriz García, Iván Fernández-Vega, Aurora Astudillo, Luis M. Quirós

**Affiliations:** ^1^Department of Morphology and Cell Biology, Universidad de Oviedo, Oviedo, Spain; ^2^Department of Functional Biology, Universidad de Oviedo, Oviedo, Spain; ^3^Department of Pathology, Hospital Universitario Central de Asturias, Oviedo, Spain; ^4^University Institute of Oncology of Asturias (IUOPA), Oviedo, Spain

**Keywords:** neuroendocrine tumor, proteoglycan, glycosaminoglycan, syndecan 2, glypican 1, chondroitin sulfate, glypican 5

## Abstract

Neuroendocrine tumors (NETs) are found throughout the body and are important as they give rise to distinct clinical syndromes. Glycosaminoglycans, in proteoglycan (PG) form or as free chains, play vital roles in every step of tumor progression. Analyzing tumor samples with different degrees of histological differentiation we determined the existence of important alterations in chondroitin sulfate (CS) chains. Analysis of the transcription of the genes responsible for the production of CS showed a decline in the expression of some genes in poorly differentiated compared to well-differentiated tumors. Using anti-CS antibodies, normal stroma was always negative whereas tumoral stroma always showed a positive staining, more intense in the highest grade carcinomas, while tumor cells were negative. Moreover, certain specific cell surface PGs experienced a drastic decrease in expression depending on tumor differentiation. Syndecan 2 levels were very low or undetectable in healthy tissues, increasing significantly in well-differentiated tumors, and decreasing in poorly differentiated NETs, and its expression levels showed a positive correlation with patient survival. Glypican 5 appeared overexpressed in high-grade tumors with epithelial differentiation, and not in those that displayed a neuroendocrine phenotype. In contrast, normal neuroendocrine cells were positive for glypican 1, displaying intense staining in cytoplasm and membrane. Low-grade NETs had increased expression of this PG, but this reduced as tumor grade increased, its expression correlating positively with patient survival. Whilst elevated glypican 1 expression has been documented in different tumors, the downregulation in high-grade tumors observed in this work suggests that this proteoglycan could be involved in cancer development in a more complex and context-dependent manner than previously thought.

## Introduction

The neuroendocrine tumors (NETs) comprise a heterogeneous group of neoplasms, which express typical characteristics of cells of the neuroendocrine system, which in itself constitutes a diffuse structure that includes several cell types, which arise in tissues derived from the embryonic neural crest, specific zones of neuroectoderm and endoderm, and have many histological similarities to neural cells, such as secretory granules, similar cellular antigens and the fact they contain the potential markers chromogranin A, synaptophysin, and neurone-specific enolase. They produce bioactive substances such as peptide hormones and biogenic amines that serve a transmitter function (1).

The incidence of NETs is two cases per 100,000 people, and they account for 0.5% of all malignancies. NETs can arise in many different parts of the body including the gastrointestinal (GI) tract, lung, pancreas, thymus, adrenal glands, ovary, and thyroid. However, approximately two-thirds of NETs are found in the GI tract, and approximately one-quarter occur in the lung, thus the two locations account for about 90% of such tumors (2). Although most NETs occur as sporadic tumors, in some cases the risk of their development is significantly increased by several genetic syndromes such as multiple endocrine neoplasia types 1 and 2, neurofibromatosis type 1, von Hippel–Lindau disease, and tuberous sclerosis (3).

Some characteristics of NETs are specific to where they are located. Nonetheless, in general and according to WHO guidelines, these neoplasms are divided into well-differentiated and poorly differentiated. Well-differentiated NETs have a characteristic organoid arrangement of the tumor cells, and in general are either low or intermediate grade with mitotic activity and Ki67 proliferative index of under 20%. In contrast, poorly differentiated NETs display a more sheetlike or diffuse architecture, higher mitotic activity and are considered high-grade in all cases (4). The highest malignancy grade tumors are further subclassified as either large cell NETs, which show anaplastic neuroendocrine growth with a more epithelial-like morphology and larger cellular size, or small cell anaplastic NETs, with smaller cell size, occasionally neural-like pseudorosettes and which are more similar to primitive neuroectodermal tumors (5).

Various studies have addressed the analysis of the differential gene expression profile in NETs and have identified alterations affecting a variable number of transcripts, ranging from around a hundred to a thousand depending on the study design and experimental analysis (6–9). Among the genes whose expression is altered there are some putative oncogenes, growth-factor related genes, transcription factors, apoptosis-related genes, and genes involved in signal transduction, cell adhesion and migration, cell growth, and cell death (6–9). Interestingly, although several of these genes have functional relationships with proteoglycans (PGs), up to now the studies performed have detected scarce changes in the expression of genes encoding these molecules.

**Figure 1:**
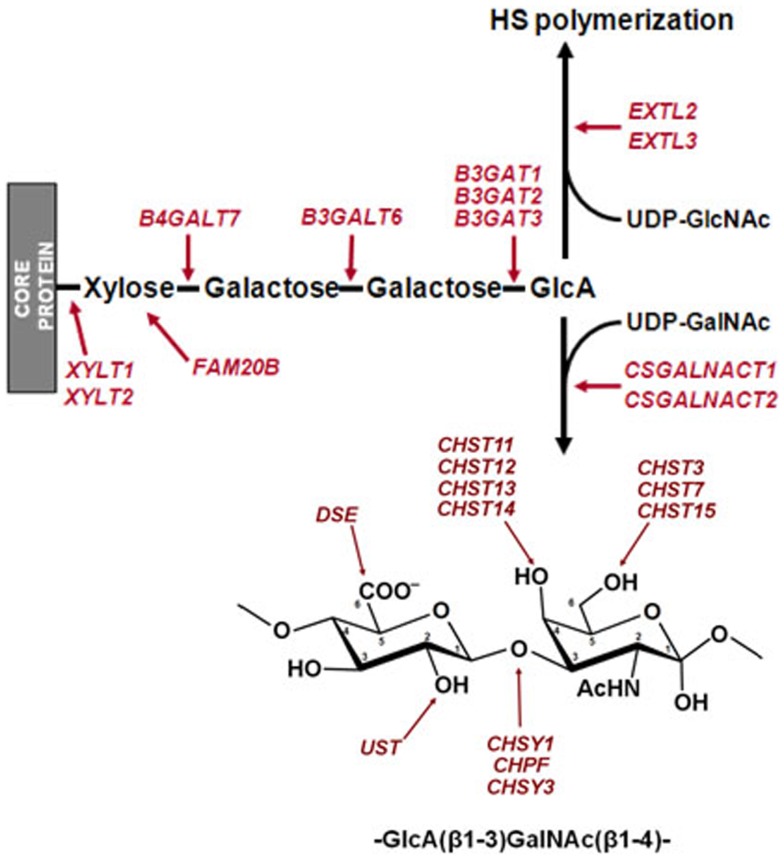
**Chondroitin sulfate and HS biosynthesis**. Biosynthesis begins with the addition of a xylose to specific serine residues acceptor of a PG core protein. Subsequently, the biosynthesis continues with the stepwise addition of two galactose units, and a GlcA. HS chain extension requires the subsequent transference of a GlcNAc residue, while the addition of a GalNAc directs the pathway toward the biosynthesis of CS. Sites of action of the enzymes involved in the initiation of the synthesis of GAG chains and in the modification of the CS disaccharide unit are indicated.

Proteoglycans are a diverse group of glycoconjugates composed of different core proteins post-translationally modified with one or more covalently attached glycosaminoglycan (GAG) chains. GAGs are linear anionic polysaccharides made up of repeating disaccharides containing acetylated amino sugar moieties and acid, mainly uronic. Differences in the type of monosaccharide in the repeating unit, as well as their sulfation, result in the various types of GAGs. Heparan sulfate (HS) and chondroitin sulfate (CS) are the two most widespread forms of sulfated GAGs, being present in all cell types and tissues at the extracellular and cellular levels. HS consists of repeating disaccharide units of *N*-acetylglucosamine (GlcNAc) and hexuronic acid residues while in CS GlcNAc is substituted by *N*-acetylgalactosamine (GalNAc) (Figure 1).

Heparan sulfate and CS synthesis occurs mainly in the Golgi apparatus and is initiated by the formation of a tetrasaccharide linkage on a cognate serine residue of the PG core, synthesized by stepwise addition of xylose, two galactose units, and a glucuronic acid (GlcA) residue. The addition of this residue is the crucial factor in determining which GAG will be synthesized; while the incorporation of GalNAc initiates CS elongation, addition of GlcNAc results in HS formation (10). Subsequently, the HS and CS chains are elongated by the addition of alternating GlcA and GlcNAc or GlcA and GalNAc residues respectively. At various positions, the molecules are modified by a series of interdependent enzymatic reactions that may include the epimerization of GlcA to iduronate (IdoA), and different sulfations of both the uronic acid and the amino sugar moieties (Figure 1) (11, 12). In HS, chain modification results in clusters of highly sulfated regions (10), while in CS it gives rise to the various different characteristic chains, CS-A, CS-C, CS-D, CS-E, and CS-B, the latter also being known as dermatan sulfate (DS) (12).

Chondroitin sulfate and HS chains are able to interact selectively with many different types of ligands, including soluble factors, membrane proteins and the extracellular matrix (ECM), lipids, and even microorganisms. HSPGs and CSPGs are mainly associated with the cell surface and the ECM and a variety of both normal and pathological functions have been ascribed to them, including cell adhesion and migration, organization of the ECM, regulation of proliferation, differentiation and morphogenesis, cytoskeleton organization, tissue repair, inflammation, vascularization, and cancer metastasis (12, 13).

Proteoglycans may play diverse roles in cancer and act as either inhibitors or promoters of tumor progression depending on the type and stage of the cancer. They have been found to be involved in several aspects of tumor biology including cell proliferation, adhesion and migration, inflammation, and angiogenesis (12–15). The expression of PGs is markedly altered during malignant transformation and tumor progression, affecting both the PG core proteins and the GAG chains (15, 16). As a result of their ability to interact with diverse molecules, PGs also regulate signaling in non-neoplastic components of the tumor, and have important roles in regulating the immune response to cancer (15, 17).

Unlike other neoplastic transformations, there is little information concerning alterations of PGs in NETs. Some studies report changes associated with malignant transformation, such as the increased expression of lumican in carcinoid compared to neuroendocrine cell carcinomas, suggesting that this PG may play a role in the slow growth of these tumors (18). The overexpression of EXTL2 in patients with MEN1 syndrome (8) has also been evidenced, as well as alterations in the levels of versican, biglycan, DSPG 3, perlecan, and CD44 in pancreatic endocrine tumors and their liver metastasis (19).

In this paper, we investigate the expression of PGs in NETs of different malignancy grade. By means of differential transcription studies we identify genes whose expression is significantly altered between tumors of low and high-grade. Subsequently, we analyze the alterations in the products of these genes, PGs or GAGs as appropriate, using immunohistochemistry, as well as their correlation with cell differentiation, grade and stage of tumor, and patient survival. Given the large number of genes involved in the biosynthesis of these complex molecules, we focus the analysis on two specific families that have been shown to be strongly involved in tumor transformation in other neoplastic processes: HSPG core proteins and enzyme encoding CS chains. The aim of the work was to detect genes whose expression may be related to tumor aggressiveness and which might be able to predict the behavior of NETs. These genes could, in the future, be useful in developing new chemical biology approaches to retarding tumor progression by modulating the deregulated biosynthetic pathways.

## Materials and Methods

### Materials

The following materials were purchased from the manufacturers indicated: RNeasy Kit and RNase-Free DNase from Qiagen (Hilden, Germany); High-Capacity cDNA Reverse Transcription Kit and PowerSYBR Green PCR Master Mix from Applied Biosystems (Foster City, CA, USA); GenElute PCR clean-up kit and 3,3′-diaminobenzidine from Sigma-Aldrich (St. Louis, MO, USA); and EnVision™ G|2 Doublestain System and EnVision FLEX Target Retrieval Solution of High pH from Dako (Glostrup, Denmark). All other chemicals were obtained from commercial sources and were of analytical grade.

The following antibodies were used in this study: rabbit anti-syndecan 2 (M-140) was from Santa Cruz Biotechnology, Inc. (Santa Cruz, CA, USA); rabbit anti-glypican 1 polyclonal antibody from Thermo Scientific (Thermo Fisher Scientific Inc., Waltham, MA, USA); rabbit anti-glypican 5 monoclonal antibody from Novus Biologicals (Littleton, CO, USA); monoclonal anti-CS clone CS-56, purchased from Sigma-Aldrich (St. Louis, MO, USA); and anti-mouse (sc-2020) and anti-rabbit (sc-2004) secondary antibodies, from Santa Cruz Biotechnology (Santa Cruz, CA, USA).

### Patients and samples

Thirty-nine patients with gastroenteropancreatic (GEP) and pulmonary neuroendocrine tumors (L-NETs) were recruited at the Biobank of the Hospital Universitario Central of Asturias between February 2000 and October 2009. Institutional review board approval in relation to guidelines on ethical procedures, and patient informed consent for research on the samples were sought and given. The diagnosis of NETs was based on morphological criteria according to the updated World Health Organization grading classification (2010). The characteristics of the patients studied (age, gender, and toxic habits) and the clinicopathological features of their tumors (WHO classification, tumor site, size, differentiation, stage, mitotic index, proliferation index, vascular invasion, lymph node status, necrosis, and presence of metastasis) were recorded. Tissues obtained from biopsies were fixed in 10% formaldehyde and paraffin embedded then cut in 4 μm thicknesses, mounted on treated slides, and stained with Hematoxylin–Eosin (H&E). The neuroendocrine phenotype was confirmed by chromogranin A and synaptophysin immunohistochemical staining. Normal tissue present in each tissue section was used as a reference.

### Tissue microarray construction

Representative tumor regions were identified in each sample and selected to make a tissue microarray containing three tissue cores from each of 31 GEP and 8 L-NETs. After 5 min at 60°C the blocks were cut into 4 μm sections in preparation for immunohistochemical techniques.

### Total RNA isolation and cDNA synthesis

To obtain the RNA, fragments of frozen tissue of between 20 and 30 mg in weight were used. Samples were homogenized using a PT 2100 Polytron (Kinematica, Inc., Bohemia, NY, USA), and RNA was isolated using the RNeasy kit, following the manufacturer’s specifications. To ensure removal of residual contaminating DNA, samples were subjected to treatment with RNase-free DNase during the purification process itself. The concentration of RNA obtained was determined spectrophotometrically by measuring absorbance using a Picodrop Microliter UV/Vis spectrophotometer (Picodrop Limited, Frozen, UK). The samples were divided into aliquots of 10 μl and used for reverse transcription reactions or stored at −20°C until further use.

cDNA synthesis was carried out using the High-Capacity cDNA Transcription Kit following the manufacturer’s specifications. The reactions were performed using an iCycler iQ PCR Thermal Cycler (BioRad, Hercules, CA, USA), using 2 μg of RNA as starting material. The reaction products were cleaned using the PCR Clean-Up GenElute kit following the manufacturer’s instructions. Finally, the aliquots containing the cDNA were diluted 1:20 with water and used for qRT-PCR assays or stored at −20°C until use.

### qRT-PCR reactions

In all cases, specific oligonucleotides were designed on different exons or exon junctions, using the program Primer 3^1^. The size of the amplicon was situated in all cases between 70 and 150 bp, ensuring wherever possible that the *T*_m_ was above 77°C. The theoretical *T*_m_ for each amplicon was determined using the program Biomath^2^.

The primer sequences were: *SDC1* (Gene ID 6382) forward 5′ CTCAGGTGCAGGTGCTTTG 3′, reverse 5′ CTGCGTGTCCTTCCAAGTG 3′; *SDC2* (Gene ID 6383) forward 5′ GATGACGATGACTACGCTTCTG 3′, reverse 5′ TGGAAGTGGTCGAGATGTTG 3′; *SDC3* (Gene ID 9672) forward 5′ CTCCTTTCCCGATGATGAAC 3′, reverse 5′ CGACTCCTGCTCGAAGTAGC 3′; *SDC4* (Gene ID 6385) forward 5′ GGCAGGAATCTGATGACTTTG 3′, reverse 5′ TCTAGAGGCACCAAGGGATG 3′; *GPC1* (Gene ID 2817) forward 5′ CATCGGGTGTGGAGAGTG 3′, reverse 5′ TGAGCGTGTCCCTGTTGTC 3′; *GPC2* (Gene ID 221914) forward 5′ CTGGGACACGACCTGGAC 3′, reverse 5′ GCCATCCAGTCATCTGCATAC 3′; *GPC3* (Gene ID 2719) forward 5′ CTGCTTCAGTCTGCAAGTATGG 3′, reverse 5′ GTGGAGTCAGGCTTGGGTAG 3′; *GPC4* (Gene ID 2239) forward 5′ AGTGTGGTCAGCGAACAGTG 3′, reverse 5′ CAAACATATCATTCAGGGATTTCTC 3′; *GPC5* (Gene ID 2262) forward 5′ GCCGCCCTGTAAGAACAC 3′, reverse 5′ TCATTCCATGCTTCTCTTTGC 3′; *GPC6* (Gene ID 10082) forward 5′ CCAGGCATAAGAAATTTGACG 3′, reverse 5′ CATGTACAGCATGCCATAGGTC 3′; *PRCAN* (Gene ID 3339) forward 5′ TGGACACATTCGTACCTTTCTG 3′, reverse 5′ CACTGCCCAGGTCGTCTC 3′; *AGRN* (Gene ID 375790) forward 5′ ACTGTGTCTGCCCGATGC 3′, reverse 5′ GACACTCGTTGCCGTATGTG 3′; *COL18A1* (Gene ID 80781) forward 5′ GTACAAGGGAGAGATTGGCTTTC 3′, reverse 5′ TTTCTCTCCTTTCAATCCGTTC 3′; *XYLT1* (Gene ID 64131) forward 5′ ACTACCCCATCAGGACAAATGA 3′, reverse 5′ CTGCTTCCGAATGAACCTTG 3′; *XYLT2* (Gene ID 64132) forward 5′ AGGGCCTGGTAGTGTGGAG 3′, reverse 5′ TGAACTGTCTGTGTCCTTGGAA 3′; *FAM20B* (Gene ID 9917) forward 5′ TCTGCAGAAGCACCGTCA 3′, reverse 5′ CAGCTGTGTCAATGATGTCCA 3′; *B4GALT7* (Gene ID 11285) forward 5′ GCGAGGACGACGAGTTCTAC 3′, reverse 5′ CAGGTGGCGAAATGTCTTGTA 3′; *B3GALT6* (Gene ID 126792) forward 5′ CACGTGGCCTTCGAGTTC 3′, reverse 5′ CCGAGAAGAAGCCCCAGTA 3′; *B3GAT1* (Gene ID 27087) forward 5′ TGGTGAATGAGGGCAAGAA 3′, reverse 5′ CTTAGGAGTCGGCCTTGGA 3′; *B3GAT2* (Gene ID 135152) forward 5′ GCTGACGACGACAACACCTA 3′, reverse 5′ CGGTGTACCAGCCAACAAC 3′; *B3GAT3* (Gene ID 26229) forward 5′ GAAGAACGTGTTTCTCGCCTAC 3′, reverse 5′ CCTCAGATCCTTCTGCCGTA 3′; *EXTL2* (Gene ID 2135) forward 5′ TGAACTGGAAACCAATGCAG 3′, reverse 5′ AGGAAATTGCTGCCAAACTG 3′; *EXTL3* (Gene ID 2137) forward 5′ CTCCGCCATGACGAAATC 3′, reverse 5′ AGTTGGAGTTGTAGAGCCAGGA 3′; *CSGALNACT1* (Gene ID 55790) forward 5′ GGAGACCCTGAACAATCCTG 3′, reverse 5′ GCCGTTTGAATTCGTGTTTG 3′; *CSGALNACT2* (Gene ID 55454) forward 5′ GCCATTGTTTATGCCAACCA 3′, reverse 5′ ATCCACCAATGGTCAGGAAA 3′; *CHSY1* (Gene ID 22856) forward 5′ GCCCAGAAATACCTGCAGAC 3′, reverse 5′ CACTACTGGAATTGGTACAGATG 3′; *CHPF* (Gene ID 79586) forward 5′ CTGGGTCGCTGCATTCTC 3′, reverse 5′ GGCACTTCGGAAATGAGG 3′; *CHSY3* (Gene ID 337876) forward 5′ CGCCGACGACGATGTCTAC 3′, reverse 5′ CCAGTCCCAGCTTTCCAAG 3′; *CHST11* (Gene ID 50515) forward 5′ CGCTGCTGGAAGTGATGA 3′, reverse 5′ CAGCAGATGTCCACACCAA 3′; *CHST12* (Gene ID 55501) forward 5′ GTAGCCGACAAATCCTTCCA 3′, reverse 5′ ACCGGTTTACCTCTGACTTGAC 3′; *CHST13* (Gene ID 166012) forward 5′ CCGGCATTTGGAAACAGA 3′, reverse 5′ TCCAGGTCATAGAGCTTCTGC 3′; *CHST14* (Gene ID 113189) forward 5′ CCACTGCCTAATGTCACCAA 3′, reverse 5′ ATGACAGGCAGAAGCACAGA 3′; *CHST15* (Gene ID 51363) forward 5′ GTGCCAGGAATAAAGTTCAACA 3′, reverse 5′ CACTGGATAAGTCCCGAGTGA 3′; *CHST3* (Gene ID 9469) forward 5′ TGCACAGCCTGAAGATGAGA 3′, reverse 5′ CAGCTTGTCTGAGACCCTTGA 3′; *CHST7* (Gene ID 56548) forward 5′ GATCCGGGTCAGTCACCA 3′, reverse 5′ GACAGATTGCCCCCACAG 3′; *DSE* (Gene ID 29940) forward 5′ GTCCAGAGGCACTTCAACATC 3′, reverse 5′ AGTCCGCAATAGCCACAGTC 3′; and *UST* (Gene ID 10090) forward 5′ ACCATGGACCACCTCCTAGTAA 3′, reverse 5′ GCTTCTCCGACAAGATTCTCA 3′.

At least four repetitions of each qRT-PCR reaction were carried out in a final volume of 10 μl, according to the manufacturer’s specifications, using 1 μl of the cDNA dilution as template, with 2 μl of primer pair mix (200 nM final concentration) and 5 μl of SYBR Green mix, contained in 96 well microtiter plates. The plates were sealed with optical film and centrifuged at 2500 rpm for 5 min before being placed in a Real-Time ABI Prism Detection System device (Applied Biosystems, Foster City, CA, USA) using the following cycling conditions: 95°C for 10 min, 40 cycles of 95°C for 15 s followed by 60°C for 60 s. Following thermal cycling and data collection steps, amplimer products were analyzed using a melt curve program (95°C for 1 min, 55°C for 1 min, then the temperature was increased by 0.5°C per cycle for 80 cycles of 10 s each). For each amplification the presence of a single peak with a *T*_m_ corresponding to that previously calculated was verified. In those cases in which the amplifications were not adequate, new primer pairs were designed. Actin was included on each plate as a control gene to compare run variation and to normalize individual gene expression.

### Data analysis

To calculate the efficiency of amplification for each gene we used the program LinRegPCR^3^, using the best correlation coefficient (considering a minimum of three points within the window of linearity) and establishing the average of all positive amplifications. At least four replicates of each reaction were carried out, with the number of replicates being increased in those reactions that showed ambiguity or dispersion of results. The values of differential expression of the genes of interest were expressed according to the equation below, as has been described previously (20).

$${\mathrm{rER}={\left(1+E\left(GOI\right)\right)}^{-\Delta C_{t}\left(GOI\right)}∕\left(1+E\left(HKG\right)\right)\;}^{-\Delta C_{t}\left(HKG\right)}$$

with Δ*C*_t_ (gene) = Δ*C*_t_ (gene, well-differentiated) − Δ*C*_t_ (gene, poorly differentiated). Here, rER is the relative expression ratio of a gene-of-interest *GOI* (relative to a housekeeping gene *HKG*).

### Immunohistochemistry

Tissue sections were dewaxed as described in the previous section. Rehydrated sections were rinsed in phosphate buffered saline (PBS) containing 1% tween-20. Sections were heated in High pH EnVision FLEX Target Retrieval Solution at 65°C for 20 min and then incubated for 20 min at room temperature in the same solution. Endogenous peroxidase activity (3% H_2_O_2_) and non-specific binding (33% fetal calf serum) were blocked and the sections were incubated overnight at 4°C with primary antibodies using a 1:100 dilution. Secondary antibodies were used at a 1:100 dilution. 3,3′-diaminobenzidine was used as a chromogen. Finally, samples were counterstained with hematoxylin, dehydrated and mounted in Entellan^®^ (Merck, Germany). The sections were studied and photographed (20× and 40× objective) under a light microscope (Nikon – Eclipse 80i).

### Immunohistochemistry assessment

The protein expression levels were evaluated by two independent observers (and a third in cases of disagreement) taking into account two parameters: immunohistochemical signal intensity (in a 0–3 point scale) and the percentage of positive cells (0–100). For statistical purposes, the tumors were divided into two groups taking the median score value for each marker as a cutoff point.

### Statistical analysis

All analyses were performed using the Statistics for Windows program (StatSoft, Inc., Tulsa, OK, USA). Mean values were compared between two samples by the Mann–Whitney *U* test and between multiple samples by the Kruskal–Wallis test. Correlations were assessed by Pearson’s correlation coefficient. *p* < 0.05 was accepted as significant.

## Results

### Analysis of differential gene expression

We investigated the differential expression of two specific families of genes involved in the biosynthesis of PGs in NETs: HSPG core proteins and CS biosynthesis enzymes. Firstly, we used qRT-PCR to perform a quantitative analysis of the differential transcription of the coding genes using eight samples of lung NETs. Poorly differentiated large cell NETs were analyzed, and compared to low-grade well-differentiated typical carcinoids. The samples corresponded to patients of both sexes, aged between 49 and 84 years.

Determining the expression of the different proteins or saccharide chains of the GAGs analyzed was performed by immunohistochemistry using a tissue array in which tumor and healthy samples of 39 patients were included (Table 1). The array included nine specimens of small intestine NETs, six pancreatic, one esophageal, eight from the colon, four from the stomach, one hepatic, two from the appendix, and eight from the lung. Twenty samples corresponded to well-differentiated and 19 to poorly differentiated tumors. Eleven of the tumor samples were in stage 1, 7 in stage 2, 14 in stage 3, and 7 in stage 4. The distribution of patients’ ages at diagnosis was as follows; 8 were aged between 39 and 49 years, 3 between 50 and 59, 16 between 60 and 69, 9 between 70 and 79, and 3 were over 80.

**Table 1:** **Patient demographics, histologic grade, and staging**.

Patient no.	Age	Sex	Location	Grade	Stage	Differentiation
1	81	F	Ileum	G3	T3	PD
2	40	F	Pancreas	G3	T4	PD
3	76	M	Esophagus	G3	T3	PD
4	44	M	Colon	G1	T3	WD
5	66	M	Ileum	G1	T3	WD
6	76	M	Stomach	G3	T3	PD
7	66	M	Ileum	G1	T3	WD
8	49	M	Liver	G1	T2	WD
9	63	M	Appendix	G1	T1	WD
10	40	F	Pancreas	G2	T4	PD
11	48	M	Pancreas	G1	T1	WD
12	61	M	Colon	G1	T2	WD
13	73	F	Colon	G1	T2	WD
14	60	F	Ileum	G1	T4	WD
15	62	M	Appendix	G1	T1	WD
16	65	M	Pancreas	G3	T3	PD
17	60	M	Colon	G3	T3	PD
18	62	F	Jejunum	G1	T3	WD
19	71	M	Ileum	G1	T3	WD
20	69	M	Stomach	G1	T3	PD
21	74	M	Stomach	G3	T2	PD
22	52	M	Jejunum	G3	T3	PD
23	55	M	Ileum	G1	T2	WD
24	70	M	Pancreas	G1	T2	WD
25	70	M	Colon	G3	T4	PD
26	77	M	Stomach	G3	T4	PD
27	64	F	Ileum	G1	T1	WD
28	42	M	Colon	G2	T4	PD
29	64	F	Colon	G3	T4	PD
30	87	F	Colon	G2	T1	PD
31	71	F	Pancreas	G1	T1	WD
32	66	M	Lung	G2	T2	PD
33	49	F	Lung	G3	T3	PD
34	67	M	Lung	G2	T1	PD
35	65	M	Lung	G3	T3	PD
36	63	M	Lung	G1	T1	WD
37	51	F	Lung	G1	T1	WD
38	84	M	Lung	G1	T1	WD
39	49	M	Lung	G1	T1	WD

*WD, well-differentiated; PD, poorly differentiated*.

### Differential expression of HSPG core proteins

Only 13 genes encode HSPG core proteins. Two gene families, syndecans and glypicans, account for most cell surface HSPGs. Respectively these families comprise 4 (SDC1–4) and 6 (GPC1–6) different proteins. The three remaining molecules are arranged in the ECM and include perlecan (PRCAN), agrin (AGRN), and collagen type 18 (COL18A1) (21).

**Figure 2:**
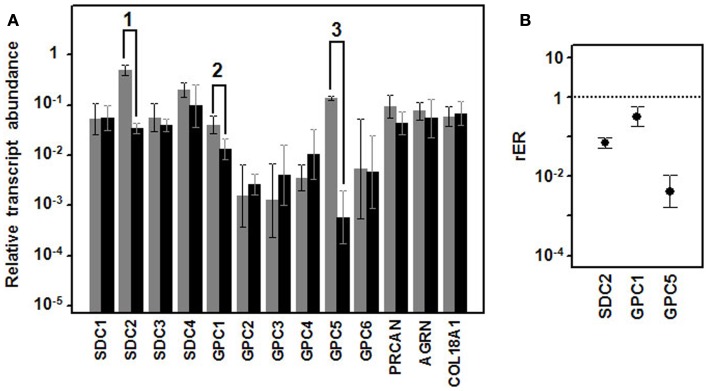
**Differential transcription of genes encoding HSPGs**. **(A)** Relative transcript abundance of mRNAs for HSPGs. Relative abundance for well-differentiated lung NETs (gray bars) and poorly differentiated lung NETs (black bars) are plotted on a log scale for each gene assayed and the spread represents standard deviation. Genes showing significant differences are indicated: 1, *p* < 0.001; 2, *p* = 0.018; 3, *p* = 0.0001. **(B)** Relative expression ratio of genes that show statistically significant differences in expression in poorly differentiated compared to well-differentiated tumors. Values on the *Y* axis are represented on a logarithmic scale.

When we analyzed the levels of transcription of these genes in well-differentiated and poorly differentiated lung tumors by means of qRT-PCR, there were no significant differences in any of the ECM PGs (Figure 2A). However, the cell surface HSPGs showed some significant differences. Within the group of syndecans, no significant differences in the levels of transcripts of isoforms 1, 3, and 4 could be detected (Figure 2A). However, syndecan 2 levels were significantly different; decreasing approximately 14-fold in high-grade tumors (Figure 2B).

**Figure 3:**
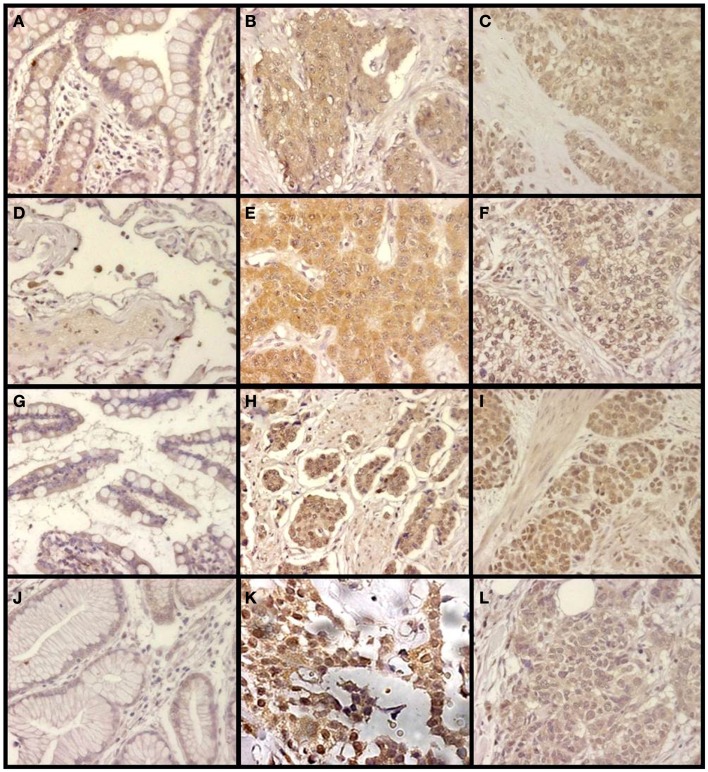
**Immunohistochemical staining of syndecan 2 in NETs**. **(A,D,G,J)** Normal tissue from colon **(A)**, lung **(D)**, small intestine **(G)**, and gastric transitional mucosa **(J)**. **(B,E,H,K)** Well-differentiated NETs from colon **(B)**, lung **(E)**, small intestine **(H)**, and pancreas **(K)**. **(C,F,I,L)** Poorly differentiated NETs from colon **(C)**, lung **(F)**, stomach **(I)**, and pancreas **(L)**. Syndecan 2 antibody marks normal epithelial cells, namely intracryptic cells, with faint cytoplasmic staining. The marking is enhanced in low degree NETs, decreasing in those NETs with the highest degree of malignancy (neuroendocrine carcinomas). Magnification 200×.

**Figure 4:**
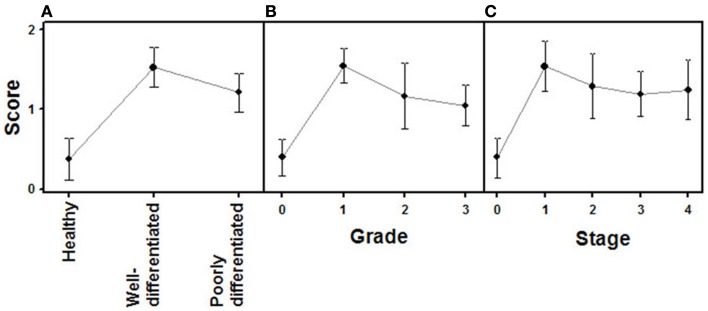
**Quantification of immunochemical staining of syndecan 2**. **(A)** Relative to the tumor differentiation. **(B)** Relative to tumor grade. **(C)** Relative to tumor stage. Semiquantitative scale from 0 to ++ was used. Vertical bars denote 0.95 confidence intervals.

Changes in the expression of syndecan 2 in NETs were evaluated by immunohistochemistry using tissue arrays with tumors of different origins, grades, and cell differentiation, as detailed above. Labeling with anti-syndecan 2 was very low or undetectable in normal tissues, increasing significantly in well-differentiated tumors, and decreasing in poorly differentiated NETs (Figure 3). Semiquantitative analysis of expression levels in normal tissues and in well- and poorly differentiated tumors allowed significant differences between them to be determined (*p* = 0.05, Kruskal–Wallis test); specifically between healthy and tumor tissues, either well- or poorly differentiated (*p* < 0.0001 and *p* = 0.003 respectively, Mann–Whitney test), although there was a reduced level of significance between the two tumor subgroups (*p* = 0.051) (Figure 4A). When the syndecan 2 semiquantitative expression levels in the different tumors were analyzed in relation to their diagnosis features, there was a significant correlation with tumor grade (*r* Spearman −0.47, *p* = 0.004) (Figure 4B) although the correlation was not significant in terms of tumor stage (*p* = 0.2) (Figure 4C). Interestingly, there was also a good positive correlation between syndecan 2 levels and patient survival (*r*_s_ = 0.6, *p* = 0.004). The cases presenting higher levels of this PG displayed better prognosis. In addition, we quantified the existence of correlations between syndecan 2 expression and tumor topography using the classical division into foregut, midgut, and hindgut (22), although no positive relationship was found (*p* = 0.85).

Analysis of the expression levels of the different glypicans showed substantial differences between them, far greater than in the syndecans, with the differences reaching up to two orders of magnitude. The qRT-PCR studies performed on well- and poorly differentiated lung NETs showed significant differences for two of the six species, glypican 1 and 5 (Figure 2). Transcripts of both genes evidenced a decreased expression in high-grade tumors, estimated at around 70% in the case of glypican 1 and about 230-fold for glypican 5.

**Figure 5:**
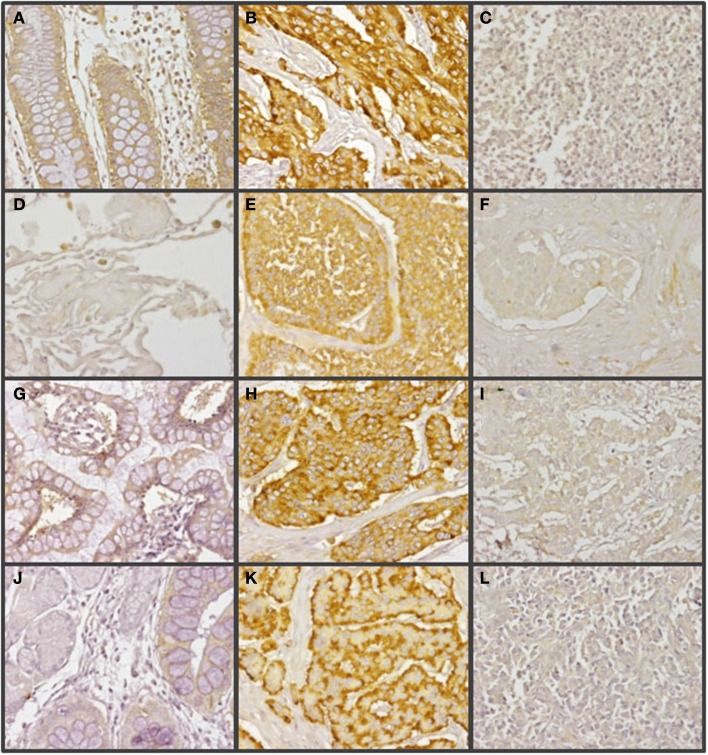
**Immunohistochemical staining of glypican 1 in NETs**. **(A,D,G,J)** Normal tissue from colon **(A)**, lung **(D)**, small intestine **(G)**, and gastric transitional mucosa **(J)**. Arrows indicate neuroendocrine cells. **(B,E,H,K)** Well-differentiated NETs from colon **(B)**, lung **(E)**, small intestine **(H)**, and pancreas **(K)**. **(C,F,I,L)** Poorly differentiated NETs from colon **(C)**, lung **(F)**, stomach **(I)**, and pancreas **(L)**. Low-grade tumors exhibited markedly increased staining compared to normal tissue in both cytoplasm and cell membrane. By contrast, high-grade tumors showed a drastic decrease in expression. Magnification 200×.

**Figure 6:**
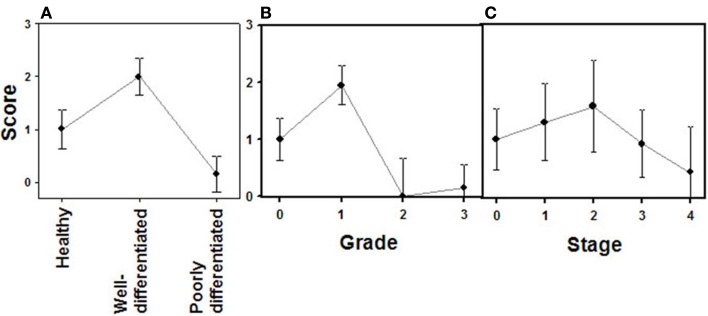
**Quantification of immunochemical staining of glypican 1**. **(A)** Relative to tumor differentiation. **(B)** Relative to tumor grade. **(C)** Relative to tumor stage. Semiquantitative scale from 0 to +++ was used. Vertical bars denote 0.95 confidence intervals.

Analogously to syndecan 2, glypican 1 expression was analyzed by immunohistochemistry using tumor tissue arrays. Expression of glypican 1 could be determined in normal tissues, but its magnitude increased strongly in well-differentiated tumors. By contrast, the expression of this protein dramatically decreased in high-grade NETs, reaching undetectable levels (Figure 5). Semiquantitative analysis of expression levels in normal tissues and in tumors showed significant differences between them (*p* < 0.0001); the differences being most marked when comparing high-grade tumors with normal tissue or well-differentiated tumors (*p* < 0.0001 in both cases), but they were also significant for the notable increase in expression when comparing normal tissue and low-grade tumors (*p* = 0.01) (Figure 6A). Furthermore, analysis of the glypican 1 expression levels in the different tumors in relation to their diagnosis features showed a significant negative correlation with tumor grade (*r*_s_ = −0.74, *p* < 0.0001), reflecting the disappearance of the expression in tumors of grade 2 or higher (Figure 6B). The correlation was more complex with respect to tumor stage (*r*_s_ = −0.33, *p* = 0.43) since expression increased in tumors up to stage 2, and then decreased progressively at higher stages (Figure 6C). Also, there was a positive correlation between the expression levels of glypican 1 and patient survival, albeit somewhat lower than the values determined for syndecan 2 (*r*_s_ = 0.54, *p* = 0.01). Furthermore, analysis of protein expression based on topography of the tumor showed no positive relationship to exist (*p* = 0.17).

**Figure 7:**
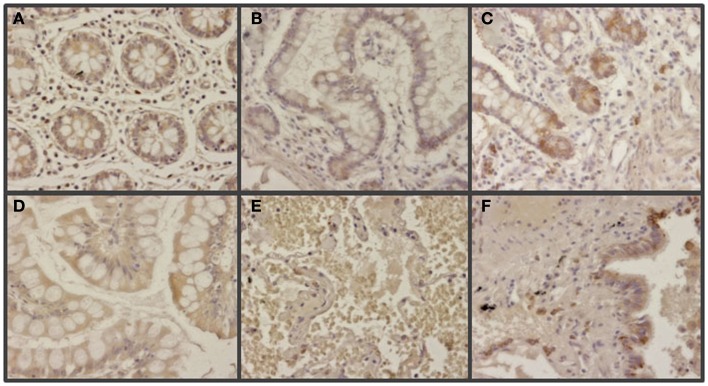
**Immunohistochemical staining of glypican 5 in normal tissues**. **(A,B,D,E)** Normal tissues not expressing GPC5 from colon **(A)**, small intestine **(B)**, gastric transitional mucosa **(D)**, and lung **(E)**. **(C,F)** Normal tissues displaying positive reaction in neuroendocrine cells from small intestine **(C)**, and lung **(F)**. Magnification 400×.

**Figure 8:**
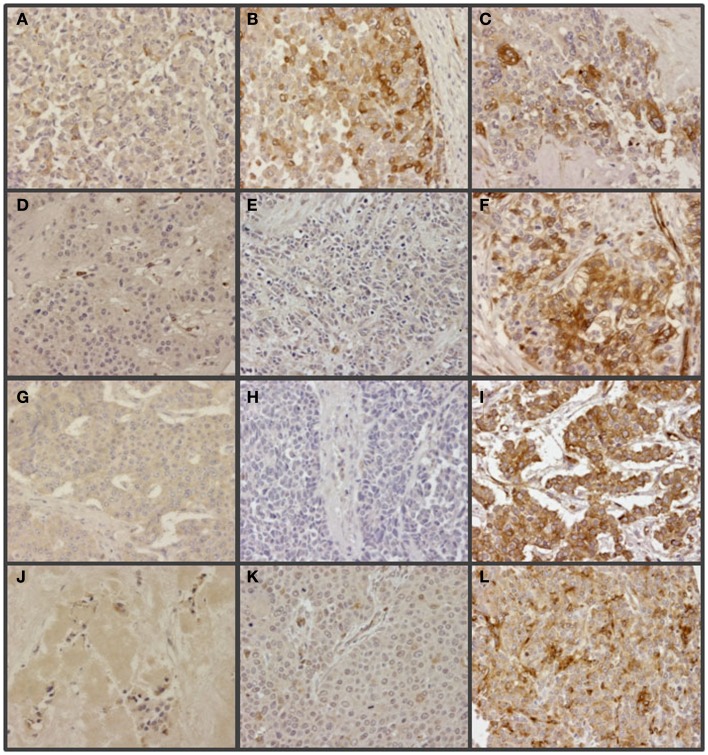
**Immunohistochemical staining of glypican 5 in NETs**. **(A,B,D,G,J)** Well-differentiated NETs; tumors not expressing GPC5 from colon **(A)**, lung **(D)**, small intestine **(G)** and pancreas **(J)**, and displaying positive reaction from colon **(B)**. **(C,E,F,H,I,K,L)** Poorly differentiated NETs; tumors showing positive reaction from colon **(C)**, lung **(F)**, stomach **(I)** and pancreas **(L)**, and not expressing GPC5 from colon **(E)**, lung **(H)** and stomach **(K)**. Magnification 200×.

**Figure 9:**
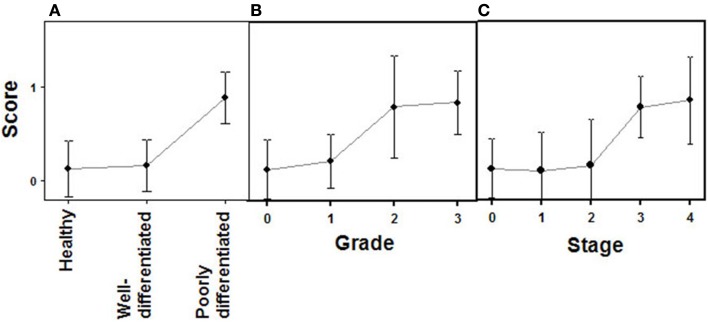
**Quantification of immunochemical staining of glypican 5**. **(A)** Relative to tumor differentiation. **(B)** Relative to tumor grade. **(C)** Relative to tumor stage. Semiquantitative scale from 0 to +++ was used. Vertical bars denote 0.95 confidence intervals.

The expression of glypican 5 was also analyzed at the protein level by immunohistochemistry. We were not able to detect positive results in the majority of normal tissues (Figure 7), although about 25% of the analyzed samples showed staining in neuroendocrine cells (Figure 7). Moreover, only 16% of low-grade tumors exhibited a positive reaction, most showing an absence of staining (Figure 8). Surprisingly, and in contrast to the data obtained for differential transcription, glypican 5 appeared overexpressed in 70% of high-grade tumors (Figure 8). High-grade tumors overexpressing the protein showed a characteristic phenotype, with epithelial differentiation, whereas those in which the molecule was not detected displayed a neuroendocrine phenotype (Figure 8). Semiquantitative analysis of expression levels allowed the observed differences to be determined as statistically significant (*p* = 0.001, Kruskal–Wallis test); specifically between well- and poorly differentiated tumors (*p* = 0.007, Mann–Whitney test) (Figure 9A). Furthermore, analysis of glypican 5 protein expression in relation to diagnosis features showed a significant level of positive correlation with tumor grade (*r*_s_ = 0.43), reflecting the increase in expression in tumors of grade 2 or higher (Figure 9B), as well as with respect to tumor stage (*r*_s_ = 0.48) since expression increased in tumors of stages 3 and 4 (Figure 9C). In contrast, there was no significant correlation between the expression levels of glypican 5 and patient survival (*r*_s_ = −0.32), and analysis of protein expression based on the topography of the tumor showed no positive relationship exists (*p* = 0.11).

### Differential expression of chondroitin sulfate chains

Chondroitin sulfate is an anionic linear polysaccharide that is implicated in cancer through its involvement in many essential aspects of cell physiology. The synthesis of CS chains initially requires an array of glycosyltransferases (GTs), which generate a tetrasaccharide glycan linker on a cognate serine residue of the PG core. This linker is shared by CS and HS chains and its sequence is integrated by xylose–galactose–galactose–GlcA (10). CS chain extension requires the subsequent transference of a GalNAc residue, followed by the sequential addition of alternating GlcA and GalNAc residues to generate a non-branched polymer. By contrast, the addition of GlcNAc rather than GalNAc directs the pathway toward the biosynthesis of HS. In this case, chain extension takes place through the sequential addition of alternating GlcA and GlcNAc residues (10, 16).

**Figure 10:**
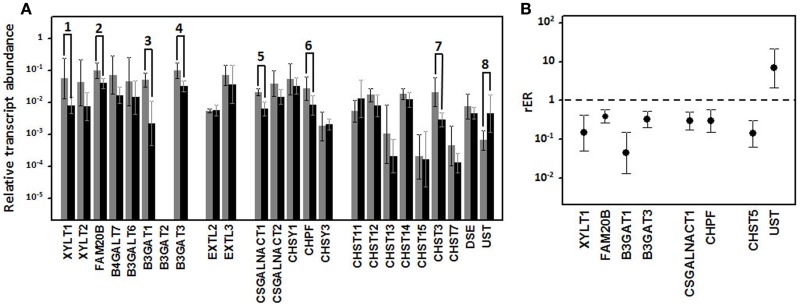
**Differential transcription of genes encoding enzymes involved in the biosynthesis of CS repeating unit**. **(A)** Relative transcript abundance of mRNAs for CS biosynthesis. Relative abundance for well-differentiated lung NETs (gray bars) and poorly differentiated lung NETs (black bars) are plotted on a log scale for each gene assayed and spread represents standard deviation. Genes showing significant differences are indicated: 1, *p* = 0.04; 2, *p* = 0.03; 3, *p* = 0.003; 4, *p* = 0.01; 5, *p* = 0.02; 6, *p* = 0.03; 7, *p* = 0.01; 8, *p* = 0.01. **(B)** Relative expression ratio of genes that show statistically significant differences in expression in poorly differentiated compared to well-differentiated tumors. Values on the *Y* axis are represented on a logarithmic scale.

We analyzed the differential transcription of seven GTs involved in the biosynthesis of the tetrasaccharide linker, *XYLT1* and *XYLT2*, responsible for the initial transfer of xylose residues, *B4GALT7* and *B3GALT6*, which encode the enzymes responsible for the sequential addition of the two residues of galactose, and *B3GAT1, B3GAT2*, and *B3GAT3*, responsible for the transfer of the GlcA residue at the end of the linker (23). It proved possible to measure amplifications of transcripts of all these genes except for *B3GAT2*. Additionally, three of these transcripts displayed a significant subexpression in poorly differentiated lung tumors compared to well-differentiated ones (Figure 10A). *XYLT1, B3GAT1*, and *B3GAT3* were downregulated around 7-, 22-, and 3-fold respectively (Figure 10B).

Alterations in the transcription of the five genes involved in the polymerization of CS chains (*CSGALNACT1, CSGALNACT2, CHSY1, CHPF*, and *CHSY3*) were also studied. Under-expression was detected in two, *CSGALNACT1* and *CHPF*, in high-grade compared to well-differentiated NETs, and in both cases the deregulation was around threefold (Figure 10).

Finally, given that the synthesis of CS chains takes place in competition with the synthesis of HS, we analyzed the two GTs responsible for transferring the first GlcNAc residue (*EXTL2* and *EXTL3*), along with the xylose kinase involved in the regulation of HS biosynthesis (*FAM20B*) (24). We were only able to detect a deregulation of the kinase, which was downregulated approximately 2.5-fold (Figure 10).

The result of the action of all GTs involved in the synthesis of CS is an unmodified chain that consists simply of repeating GalNAc–GlcA units. As the chain polymerizes, it undergoes a series of modifications that include the epimerization of GlcA in CS chains, which results in DS chains, catalyzed by DSE; the addition of sulfate groups at C2 of IdoA residue of DS, catalyzed by chondroitin uronosyl sulfotransferase (UST); sulfation at C4 of GalNAc, catalyzed by different isoenzymes with specificity for CS or DS chains (CHS11, CHS12, CHS13, and CHS14); addition of sulfate at C6 of GalNAc, also catalyzed by different isoenzymes (CHS3 and CHS7); and sulfation at C6 can also occur in pre-sulfated residues catalyzed by a *N*-acetylgalactosamine 4-sulfate 6-*O*-sulfotransferase (CHS15) (Figure 1) (23). The analysis of the differential transcription of these genes in high-grade compared to low-grade tumors showed significant differences for only two of them: CHST3, whose transcription was downregulated around seven times, and UST, which showed a sevenfold overexpression (Figure 10).

**Figure 11:**
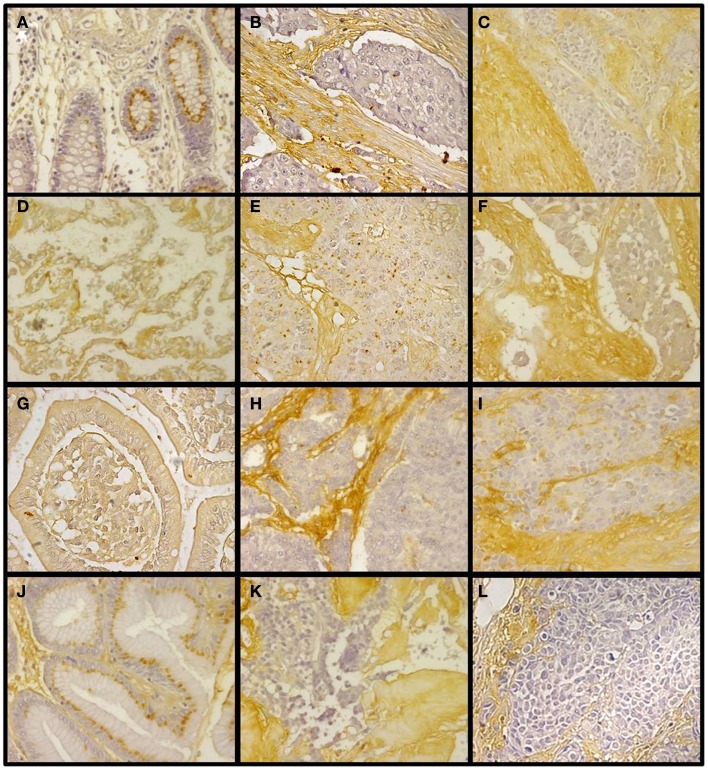
**Immunohistochemical staining of CS in NETs**. **(A,D,G,J)** Normal tissue from colon **(A)**, lung **(D)**, small intestine **(G)**, and gastric transitional mucosa **(J)**. **(B,E,H,K)** Well-differentiated NETs from colon **(B)**, lung **(E)**, small intestine **(H)**, and pancreas **(K)**. **(C,F,I,L)** Poorly differentiated NETs from colon **(C)**, lung **(F)**, stomach **(I)**, and pancreas **(L)**. Normal tissue samples displayed faint stromal staining, also present in isolated intestinal epithelial cells **(A,D,G,J)**; well-differentiated NETs showed focal stromal staining **(B,E,H,K)**, clearly enhanced in the cases of poorly differentiated tumors **(C,F,I,L)**. Magnification 400×.

**Figure 12:**
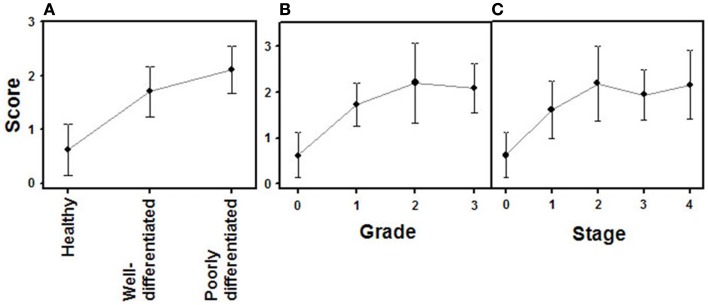
**Quantification of immunochemical staining of CS**. **(A)** Relative to tumor differentiation. **(B)** Relative to tumor grade. **(C)** Relative to tumor stage. Semiquantitative scale from 0 to +++ was used. Vertical bars denote 0.95 confidence intervals.

The combined action of all the above genes results in CS chains with specific patterns of sulfation. Changes in the expression of CS chains in NETs were evaluated by immunohistochemistry using tissue arrays, as indicated above, and monoclonal anti-CS antibodies. Antibodies applied to normal tissue sections showed faint stromal staining, which was also present in isolated intestinal epithelial cells. However, in well-differentiated NETs we found focal stromal staining, which was clearly enhanced in the cases of the highest grades (Figure 11). Semiquantitative analysis of expression levels in normal tissues and in well- and poorly differentiated tumors allowed the existence of significant differences between healthy and well-differentiated NETs (*p* < 0.01) and healthy and poorly differentiated NETs (*p* < 0.0001), but not between high-grade and low-grade tumors (*p* = 0.46) to be determined (Figure 12A). In the tumor samples there were no significant correlations between level of expression of CS and tumor grade or stage (*p* = 0.5 and *p* = 0.34 respectively), although these correlations did become significant when expression levels in normal tissues were also considered (*p* = 0.0002 in both cases) (Figures 12B,C). Moreover, no positive correlation was observed between the expression of CS and either patient survival (*p* = 0.6) or location of the tumor (*p* = 0.97).

## Discussion

Unlike other tumor types such as lung cancer or breast cancer, which are defined by their tissue of origin, NETs arise from a cell type, and may have originated in many parts of the body, although GI and lung tumors account for around 90% of all cases. Although some of the clinical and pathological features of these tumors are specific to the organ of origin, other attributes are shared by all these neoplasms and there are many similarities among NETs throughout the body (4). Taking into account their characteristics, NETs can be classified based on different criteria, such as neoplasm grade, presence of associated secretory symptoms, and anatomical site of origin or histology. The histological classification is widely accepted and divides the tumors into well- and poorly differentiated. On the other hand, classification based on anatomical location has usually divided tumors into foregut, midgut, and hindgut, but raises some controversies, such as the heterogeneity in the 5-year survival rates of the foregut neoplasms, since cancers of the biliary system and pancreas are included in this group (4, 22). Because of their diversity, NETs pose a major clinical challenge, both in diagnosis and treatment if surgery is not feasible (22).

Proteoglycans are critical in regulating the activity of many signaling pathways, as well as cell–microenvironment interactions. The development of cancer typically involves distinct stages, including transformation, invasion and metastasis, and as a result of their diverse functions, PGs and their GAG chains have been found to be involved in tumorigenesis in several cancers, including breast, lung, brain, pancreatic, and colorectal. Many studies suggest that PGs regulate multiple oncogenic pathways in tumor cells and promote critical tumor–microenvironment interactions, such that these molecules are potentially important tumor markers and therapeutic targets (15).

More than 50 members of the PG family have been characterized and furthermore, many dozens of different enzymes have been found to be responsible for the synthesis and editing of GAG chains, including GTs, deacetylases, *N*- and *O*-sulfotransferases, epimerases, sulfatases, kinases, or hydrolases, besides enzymes involved in the synthesis and transport of precursors (12, 23, 24). We focused this study on two specific groups of genes whose alterations are particularly relevant in other tumor models previously described; the HSPG core proteins and the enzymes involved in the biosynthesis of CS chains.

In human cells, there are 13 genes encoding full-time HSPGs, although a few more may appear as part-time ones (10, 25). Of the three ECM proteins studied, none showed alterations between high- and low-grade lung NETs. In several tumor models alterations in some of these genes have been described, particularly in perlecan, which is fundamental for the maintenance of basement membrane homeostasis (26), suggesting that its alteration could play an important role in NET progression. Nevertheless, we must consider that this differential transcription study analyses differences in expression between well- and poorly differentiated tumors, and not those that may have occurred as a consequence of tumor transformation. However, in some tumors alterations in expression levels of perlecan have been described, not only in relation to normal tissue, but also depending on the stage of tumor progression (27).

Two gene families, syndecans and glypicans, account for most cell surface HSPGs. Syndecans are a family of type 1 transmembrane proteins that comprises four members (SDC1–4). In the syndecans group, only *SDC2* transcripts appeared to be downregulated, approximately 14-fold in high-grade-relative to well-differentiated tumors. At the level of protein expression, analysis of various tumors by immunohistochemistry revealed an overexpression in low-grade tumors compared to normal tissues, which showed very low or undetectable levels, and a decrease in high-grade tumors, although apparently less intense than that determined for mRNA. Syndecans act as cell surface receptors but, in contrast to other types of receptors that interact with a limited number of ligands, they can interact with many extracellular molecules, including different types of soluble and insoluble proteins, lipids, and even microorganisms; this allows them to play a dual role as both adhesion receptors and docking receptors (28).

Overexpression of SDC2 has been previously described in some malignances, including Lewis lung carcinoma, ovarian and brain tumors, mesothelioma, osteosarcoma, and colon cancer (29–33). In this study, SDC2 expression was not influenced by the location of the tumor (foregut, midgut, or hindgut), suggesting that it is a common feature of these neoplasms throughout the body and that their size or spread do not significantly influence the expression of this protein. It has been proposed that this molecule plays an important role in promoting tumorigenesis and angiogenesis. As such, in colorectal cancer and Lewis lung carcinoma SDC2 can promote tumorigenesis by acting on the regulation of cell adhesion, spreading, and cytoskeletal organization (29, 30). In addition, its role in angiogenesis is supported by various data such as its high expression in the microvasculature of mouse glioma tumors (31), or its essential role in the sprouting of new blood vessels during zebrafish embryogenesis (34) and in many steps of human “*in vitro*” neovascularization (35). Interestingly, however, in the tumors analyzed in this work there was a good positive correlation between SDC2 levels and patient survival, data that has also been described in esophageal squamous cell carcinoma, prostate cancer, and pancreatic ductal adenocarcinoma, leading to the proposal that the expression levels of this molecule are of interest for diagnosis (36–38). These observations can be related to data suggesting a role for this PG as a metastasis inhibitor; thus a highly metastatic variant of Lewis lung carcinoma cells displays low SDC2 expression, and this molecule is able to suppress activation of matrix metalloproteinase-2 (39); Furthermore, it has been described that osteosarcoma cells that overexpress SDC2 are restricted in their migration and also exhibit enhanced apoptosis *in vitro* (40). Therefore it would appear that the role that SDC2 plays is complex and depends on the type of tumor and microenvironmental factors.

The glypicans constitute a six-member family of cell surface PGs that are glycosylphosphatidylinositol (GPI)-linked to the cell membrane. Their expression is cell type and developmental-stage-specific and they are involved in fundamental biological processes including cell division, interactions with ECM, differentiation, and morphogenesis (33). At the level of signaling, they are implicated in the regulation of several pathways, where they can either stimulate or inhibit activity depending on the biological context. These pathways include Wnts, Hedgehogs, FGF, bone morphogenic protein, and insulin-like growth factor (41). The analysis of the expression of the six protein isoforms in well- and poorly differentiated lung tumors by means of qRT-PCR showed approximately threefold derregulations for GPC1 and about 230-fold for GPC5; none of the other species displayed significant differences. It is of note that GPC1, 3, and 5 have been implicated in the tumorigenic process, mostly affecting growth-factor signaling and cell proliferation.

In adults, GPC5 is primarily expressed in the brain, and it is also detected in fetal brain, lung, and liver. In well-differentiated NETs, GPC5 mRNA shows the highest level of transcription relative to the other glypican isoforms, but this transcription declines dramatically in poorly differentiated tumors. Surprisingly, analysis of the expression of GPC5 at the protein level, performed by immunohistochemistry, revealed that only a small proportion of normal tissues and well-differentiated tumors showed a positive reaction, whereas the protein was overexpressed by 70% of high-grade tumors. Furthermore, there were some phenotypic differences between poorly differentiated tumors expressing the protein, which displayed epithelial differentiation, and those that did not, which showed a neuroendocrine phenotype. Previous studies have reported low correlations between mRNA levels and protein expression in many genes, suggesting different mechanisms are responsible for the observed differences in the quantitative relation between transcriptome and translatome (42). In studies of lung adenocarcinoma it has been described that the majority of genes analyzed (16/21) the protein/mRNA correlation did not differ between stages 1 and 3 tumors, indicating a similar regulatory relationship between mRNA and protein. However, some genes (5/21) did show significant differences in correlation coefficients between stages 1 and 3 lung adenocarcinomas, and for 3 of these the change was because of a relative increase in protein expression in stage 3 tumors (43). In addition, other studies in various tumors such as bladder cancer, show good correlation between transcript alterations and protein levels, albeit with a few exceptions, which have shown disagreement between transcript alteration and protein alteration (44). These data indicate that in the expression of GPC5 in NETs additional post-transcriptional mechanisms, such as protein translation, degradation, inhibition by feedback loops, or miRNA regulation may be involved. The data obtained in the current work at the protein level demonstrating that high-grade tumors display epithelial differentiation are consistent with those previously described for lymphoma, where overexpression of this gene may contribute to the development and progression of the tumor (45), and rhabdomyosarcoma (primarily pediatric sarcomas resembling developing skeletal muscle) where GPC5 is upregulated and strongly associated with the appearance of and increase in cell proliferation (46). Our data show GPC5 protein expression to be correlated with tumor grade, increasing in tumors of grade 2 or higher, and with tumor stage, increasing in tumors of stages 3 and 4, but not with patient survival or the topography of the tumor.

With regard to GPC1, immunohistochemical studies show strong expression levels in well-differentiated NETs, which decreases drastically and disappears altogether in poorly differentiated ones. As with SDC2 and GPC5, expression was not influenced by the location of the tumor, suggesting that this is another common feature shared by all neoplasms, wherever they are in the body. However, when transcription levels of the gene were analyzed, poorly differentiated tumors displayed mRNA levels of around 30% of those present in well-differentiated NETs. This discrepancy again suggests the existence of other, overlapping, levels of regulation, possibly at the level of translation; as such, the existence of translation-level regulation has been described in some genes involved in the biosynthesis of GAGs and PGs (47–49). GPC1 expression correlated well with tumor grade, and showed some degree of positive relationship with patient survival, which suggests it has possible uses as a prognosis factor.

GPC1 overexpression has been described in certain tumors, although with some peculiarities depending on the neoplasia. This isoform shows increased expression in gliomas, where it acts to enhance FGF signaling and mitogenesis (50); in pancreatic cancer GPC1 is strongly expressed both by the cancer cells and the adjacent fibroblasts, whereas its expression is low in the normal pancreas (51); in breast cancer GPC1 expression is strong in high-grade tumors (52), whilst in prostate cancer high expression occurs in tumor stroma although it disappears in tumor epithelial cells (53).

Although GPC3 is the species for which most expression alterations in tumors have been described, it does not appear to undergo alterations in NETs in relation to the degree of cell differentiation. Unlike isoforms 1 and 5, whose alterations involve increased protein expressions associated with carcinogenesis, variations in GPC3 expression levels vary greatly depending on the type of neoplasm (33, 41). Downregulation of GPC3 has been described in many tumor types, including breast, lung, gastric, and ovarian cancers and mesothelioma (41). However, in tumors originating from tissues that only express GPC3 in the embryo, its expression tends to appear with malignant transformation (41). The effects of loss of GPC3 on tumor development are compatible with its function as a tumor suppressor, since this molecule is an inhibitor of cell proliferation and can induce apoptosis (54). However, GPC3 overexpression can act as an oncogene in some tumors, such as hepatocellular carcinomas (55). It is worth noting that, in this work, we report a decrease in the expression of GPC1 and an increase in GPC5 with increasing tumor grade, suggesting that the involvement of these molecules in cancer, as in the case of GPC3, may range from tumor suppressors to oncogenes depending on tumoral context.

Most, if not all, HSPGs can be hybrid molecules, carrying both HS and CS side chains (21), besides specific CSPG molecules that can be located in the extracellular space, at the cell surface and also intracellularly (12). CS chains regulate key cellular processes, including ECM assembly, proliferation, apoptosis, migration, adhesion, invasion, and metastasis, and their molecular structures are among the most widely and most frequently altered in cancer (12, 56). Both HS and CS chain biosynthesis begins with the generation of a tetrasaccharide linkage on specific acceptor serine residues of a PG core protein. At this point, there are two competing paths: toward the synthesis of CS or HS, depending on the addition of a GalNAc or a GlcNAc residue. Finally, CS chains are elongated by the addition of alternating GlcA and GalNAc residues. Analyzing the differential transcription of all genes involved in this process in well-differentiated tumors compared to poorly differentiated, we found downregulation of *XYLT1*, responsible for the initial transfer of xylose residue, *B3GAT1*, and *B3GAT3*, responsible for the transfer of the first GlcA, *CSGALNACT1*, one of the genes that directs biosynthesis toward CS chains, and *CHPF*, involved in CS polymerization. Taken together, these data indicate a decrease in the synthesis of CS chains in poorly differentiated tumors. This conclusion is reinforced by the lack of alteration in the enzymes responsible for the initiation of the synthesis of HS *EXTL2* and *EXTL3*, which acts in competition with CS biosynthesis. In addition, during synthesis of the linkage region, a transient phosphorylation of the xyl residue occurs, catalyzed by a xylose kinase (encoded by *FAM20B*), which is dephosphorylated before subsequent polymerization. If persistent xylose phosphorylation occurs, the transference of a GlcNAc residue, catalyzed by EXTL2, terminates HS chain elongation (24). *FAM20B* also undergoes a 2.5-fold deregulation, which reinforces this proposal.

With regard to enzymes responsible for the fine structure of CS chains, there were no significant changes in any of the 4-*O* sulfotransferases or in GlcA epimerase. By contrast, *CHST3*, responsible for sulfation at C-6 of GalNAc, experienced a significant deregulation. Although the other two enzymes capable of transferring a sulfate to this position, encoded by *CHST15* and *CHST7*, showed no alteration in their levels in high-grade tumors, *CHST3* downregulation may have greater relevance when considering that *CHST15* and *CHST7* transcript levels are about two orders of magnitude lower than *CHST3*, which could significantly influence the sulfation of CS in C-6. Finally, the addition of sulfate groups of uronic acid residues, catalyzed by chondroitin UST at C2 increased sevenfold.

To evaluate the expression of CS chains in NETs by immunohistochemistry using tissue arrays, we used a monoclonal anti-CS antibody CS-56. This antibody reacts preferentially with CS-D (sulfated at C-2 and C-6), but is also able to recognize other types of structures, including CS-A, -B, -C, and -E (57). Normal tissue showed a mild staining, also present in isolated intestinal epithelial cells. In contrast, well-differentiated NETs showed focal stromal staining that was enhanced in poorly differentiated tumors. However, it is necessary to consider that the differential transcription studies were performed using tumor tissue samples with the greatest homogeneity and cellularity possible, hence their results essentially reflect events that take place in the tumor cells and not in the stroma. In the same way, the existence of stromal reaction with abnormal expression of PGs and increased concentration of CS in numerous tumors has been described (12, 58, 59).

Finally, a result of note common to all sections analyzed in this work was the absence of significant correlations between the expression of these molecules and the anatomic location of the tumors, suggesting that the alterations described are common features of all NETs.

## Author Contributions

Olivia García-Suárez carried out the histochemistry. Beatriz García performed the qPCR experiments and PCR data analyses. Iván Fernández-Vega contributed to sample and array preparation. Aurora Astudillo supervised sample preparation, the compilation of patient information as well as the histochemical analyses. Luis M. Quirós co-ordinated the study and drafted the manuscript. All authors have read and approved the final manuscript.

## Conflict of Interest Statement

The authors declare that the research was conducted in the absence of any commercial or financial relationships that could be construed as a potential conflict of interest.

## Footnotes

^1^http://biotools.umassmed.edu/bioapps/primer3_www.cgi

^2^http://www.promega.com/techserv/tools/biomath/calc11.htm

^3^http://www.gene-quantification.de/download.html
